# The plant–pathogen haustorial interface at a glance

**DOI:** 10.1242/jcs.237958

**Published:** 2020-03-10

**Authors:** Tolga O. Bozkurt, Sophien Kamoun

**Affiliations:** 1 Imperial College London, Department of Life Sciences, London, UK; 2 The Sainsbury Laboratory, University of East Anglia, Norwich Research Park, Norwich NR4 7UH, UK

**Keywords:** Defense-related autophagy, Effector translocation, Extrahaustorial membrane, Haustorium, Host–pathogen interface, Plant–pathogen interaction

## Abstract

Many filamentous pathogens invade plant cells through specialized hyphae called haustoria. These infection structures are enveloped by a newly synthesized plant-derived membrane called the extrahaustorial membrane (EHM). This specialized membrane is the ultimate interface between the plant and pathogen, and is key to the success or failure of infection. Strikingly, the EHM is reminiscent of host-derived membrane interfaces that engulf intracellular metazoan parasites. These perimicrobial interfaces are critical sites where pathogens facilitate nutrient uptake and deploy virulence factors to disarm cellular defenses mounted by their hosts. Although the mechanisms underlying the biogenesis and functions of these host–microbe interfaces are poorly understood, recent studies have provided new insights into the cellular and molecular mechanisms involved. In this Cell Science at a Glance and the accompanying poster, we summarize these recent advances with a specific focus on the haustorial interfaces associated with filamentous plant pathogens. We highlight the progress in the field that fundamentally underpin this research topic. Furthermore, we relate our knowledge of plant–filamentous pathogen interfaces to those generated by other plant-associated organisms. Finally, we compare the similarities between host–pathogen interfaces in plants and animals, and emphasize the key questions in this research area.

## Abstract (precis)

**Summary:** A discussion of the distinct host cell transport routes that underpin the biogenesis and function of plant–pathogen interfaces.

## Introduction

Plant pathogens produce specialized cellular structures that invade host cells but remain enveloped by host-derived membranes. One such structure is the haustorium produced by many species of fungi and oomycetes (herein referred to as filamentous pathogens) (Panstruga and Dodds, 2009). Haustoria form tight membrane interfaces between these plant pathogens and their invaded host cells (haustoriated cells) (Bozkurt et al., 2015, 2014; Whisson et al., 2016; Bozkurt et al., 2011) and resemble to some degree host-derived membrane interfaces that engulf intracellular metazoan parasites (Haldar et al., 2006). These interfaces are a key cellular site of the tug-of-war between pathogens and their hosts, which ends in either host colonization or pathogen arrest.

**Figure:**
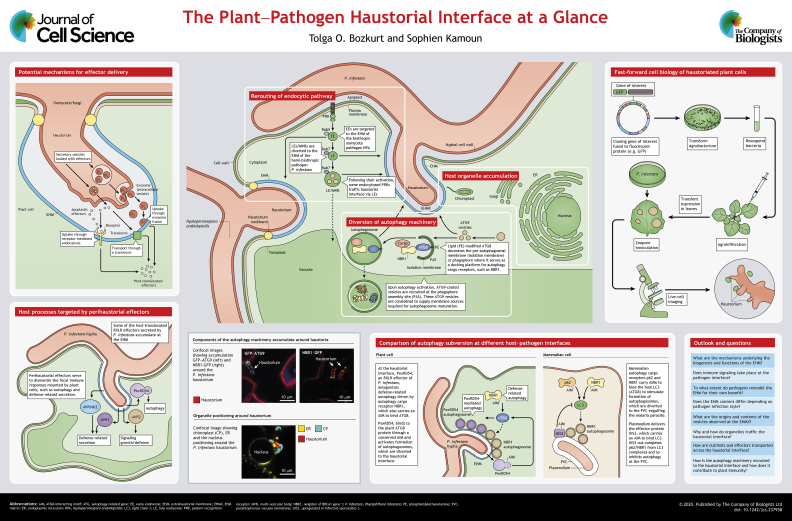

Our understanding of the biogenesis and functions of plant–pathogen interfaces remains somewhat superficial, but recent advances have yielded new insights into cellular and molecular mechanisms. Here, we summarize this new knowledge with a focus on the haustorial interfaces associated with filamentous pathogens. We emphasize the two major questions that underpin this research topic, how are plant–pathogen membrane interfaces formed and what are the functions of haustoria? We also relate our understanding of plant-filamentous pathogen interfaces to other interfaces generated by other plant-associated organisms (see Box 1).

**Box 1. Parasitic plants form haustoria too!**

Parasitic plants produce specialized structures – also known as haustoria –to acquire water and nutrients from their hosts (Yoshida and Shirasu, 2012; Kokla and Melnyk, 2018). Although the haustoria of parasitic plants appear to be functionally analogous to those of filamentous pathogens, they result in very distinct interfaces with the host plants. In contrast to filamentous pathogens, haustoria of parasitic plants are multicellular organs that differentiate from stems and roots to penetrate host tissue and directly connect the parasite vasculature to that of its host. Haustoria thus enable the parasite to siphon nutrients and create an interface that facilitates bidirectional exchanges of macromolecules. Among the trafficking trans-species molecules are various types of RNA, including mRNAs and microRNAs (miRNAs). Although the precise functions of these RNAs are still being elucidated, trans-species RNA produced by the parasite mediate the cleavage of host mRNAs to modulate host gene expression presumably to the parasite's advantage (Shahid et al., 2018; Johnson and Axtell, 2019).

## The haustorial interface

Haustoria are thought to facilitate exchange of macromolecules between the host and the pathogen. These specialized infection compartments are typically separated from the host cytoplasm through a newly synthesized plant-derived membrane called the extrahaustorial membrane (EHM) (Bozkurt et al., 2015, 2014; Whisson et al., 2016; Bozkurt et al., 2011). The haustorial interface is demarcated on one side by the EHM and on the other by the pathogen membrane and cell wall that surround the haustorium (see poster). These are separated by an extracellular matrix called the extrahaustorial matrix (EHMX) (Peresypkin et al., 1979; Baka, 2002). The number of haustoria per haustoriated host cell varies depending on the pathogen. Both oomycetes and fungi can form multiple haustoria in an individual plant cell (Bindschedler et al., 2009). However, unlike in oomycetes, the fungal haustorium is typically a separate cell that has its own nucleus with a haustorial neckband marking the cell border. Unlike intracellular hypha that can grow relentlessly and invade neighboring cells, haustoria remain restricted to infected host cells and are a terminal hyphal state.

Phylogenetically unrelated filamentous pathogens, such as the oomycetes, powdery mildew ascomycetes and rust basidiomycetes have evolved the haustorial lifestyle independently (Latijnhouwers et al., 2003). Despite their common physiological identity, the precise molecular features of the haustorial interfaces produced by these different classes of filamentous pathogens are unlikely to be the same even though some common features have been noted, as described herein.

Haustoria are not limited to filamentous pathogens. Strikingly, parasitic plants also form haustoria to tap into nutrient resources of their host plants (see Box 1). The convergent evolution of haustoria in divergent filamentous pathogens and parasitic plants further points to their importance for successful parasitism on plants.

## Molecular traffic across the haustorial interface

The specific accumulation of a sugar transporter at fungal haustoria provided the evidence that haustoria can mediate nutrient uptake (Mendgen and Nass, 1988; Hahn et al., 1997; Voegele et al., 2001). However, direct evidence for the channeling of nutrients through the haustorial interface is still generally lacking. More recently, haustorial interfaces have emerged as delivery sites of pathogen-encoded virulence factors known as effectors (Kemen et al., 2005; Whisson et al., 2007; Wang et al., 2018); these not only include proteins, but also various species of RNAs with immunomodulatory functions produced by parasitic plants (Box 1; see poster). Fungal and filamentous pathogens also appear to deploy small RNAs inside their host cells to subvert host immunity (Sperschneider et al., 2018preprint; Dunker et al., 2019, preprint). However, it is not clear whether these nucleic acids are specifically transported through the haustorial interface. More importantly, the inter-organismal transport mechanisms across the haustorial interface remain uncharacterized. One possible transport mechanism could employ extracellular vesicles (EVs), which have established roles in cell-to-cell communication (see poster). Supporting this view, EVs with unknown identity have been observed at the EHMX during fungal invasion of plant cells (Micali et al., 2011). Furthermore, the finding that both pathogens and plant can discharge EVs with immunomodulatory functions (Bahar et al., 2016; Wang et al., 2017a,b; Cai et al., 2018; Baldrich et al., 2019) has sparked renewed interest in dissecting the contents and functions of the EVs deployed at the haustorial interface.

## Effector delivery through the EHM

Specialized filamentous pathogens deliver effectors inside host cells to downregulate plant immunity and promote infection (Bozkurt et al., 2012; Thordal-Christensen et al., 2018). However, how these effectors enter plant cells remains a mystery. The majority of host-translocated oomycete effectors carry a conserved amino acid region defined by the RXLR motif that follows the N-terminal secretion signal (Whisson et al., 2007). The RXLR domain is dispensable for effector activities inside the host cells and mediates host translocation, similar to the PEXEL element found in plasmodium effectors (Hiller et al., 2004; Bozkurt et al., 2012). Like the PEXEL element, the RXLR motif undergoes proteolytic cleavage inside the parasite, with mature effectors lacking the motif (Boddey et al., 2016; Wawra et al., 2017). However, the precise mechanism by which the RXLR domain mediates effector translocation is still under debate, as the proposed models lack conclusive experimental evidence (Petre and Kamoun, 2014). For instance, the hypothesis that effector uptake takes place via binding of the RXLR motif to plant-derived phospholipids at the plant cell surface contradicts the finding that the RXLR motif is cleaved inside the pathogen prior to secretion (Wawra et al., 2017). In addition, more recent findings point to non-conventional secretory routes for host-translocation of RXLR effectors through the haustorial interface (Wang et al., 2017a,b; Wang et al., 2018).

The process of effector delivery is likely to have emerged multiple times throughout the evolution of filamentous pathogens. Unlike what is seen for oomycetes, conserved cell entry motifs and domains have not been identified in fungal effectors (Petre and Kamoun, 2014). The process is likely to be different in fungi as fungal haustoria are separate cells with nuclei and other organelles. (Petre and Kamoun, 2014). Because the fungal haustorium cell is accommodated inside the plant cell, it is assumed that the majority of the proteins secreted by fungal haustorium are either host-translocated or function at the EHMX. The translocation of effectors through the haustorial interface could possibly occur by (1) receptor-mediated endocytosis, (2) fusion of EVs loaded with effectors, or (3) through active transport facilitated by a pathogen-encoded translocon (see poster), as is the case in the apicomplexan parasite *Plasmodium* (Matthews et al., 2019).

## EHM composition is different from the plasma membrane

One striking observation, originally made over a decade ago, is that the protein and lipid composition of the EHM contrasts sharply with that of the adjacent plasma membrane. Most of the proteins embedded in the plasma membrane, such as surface immune receptors, are excluded from the EHM (Koh et al., 2005; Micali et al., 2011; Lu et al., 2012). The few exceptions include the membrane-associated remorin protein REM1.3 and the vesicle fusion protein SYT1. Particularly, REM1.3 and SYT1 are exclusively localized to discrete micro-domains along the EHM, revealing that the EHM is not a uniform interface (Bozkurt et al., 2014) (see poster). Furthermore, the plasma membrane-localized pattern recognition receptor FLS2 is found to label the EHM of the oomycete pathogen *Hyaloperonospora arabidopsidis* (HPA) but not that of *Phytophthora infestans*. This indicates that the EHM composition varies depending on the pathosystem, although experimental differences between systems cannot be totally ruled out (Lu et al., 2012).

In some cases, the EHM remains isolated from the rest of the cytosol through encasements that are formed by defense-related focal deployment of the plant cell wall material callose (Micali et al., 2011; Caillaud et al., 2014). In contrast, haustoria of the oomycete pathogen *P. infestans* are generally not fully encased, with only 20% of the haustoria showing a ‘collar’ of callose around the haustorial neck (Bozkurt et al., 2014), indicating that pathogens can further modify the perihaustorial niche or that the host prevents encasement. It is conceivable that the callose encasements contribute to the overall defense mechanisms by preventing pathogen access to host resources and defense systems. However, the degree to which pathogens suppress haustoria-related defense processes, such as callose encasements, is not understood.

## Rerouting of host-endocytic pathways to the haustorial interface

How infected plant cells selectively sort proteins into the EHM is poorly understood. The emerging paradigm is that diverse vesicular pathways may converge toward the EHM to generate a mosaic membrane interface (see poster). The EHM appears to accommodate proteins from diverse origins, including the plasma membrane, the vacuolar membrane, endocytic vesicles, plasmodesmata and the ER (Wang et al., 2009; Bozkurt et al., 2014, 2015; Caillaud et al., 2014; Inada et al., 2016; Kwaaitaal et al., 2017; Dagdas et al., 2018). It is plausible that redirection of multiple stress-related transport routes accounts for EHM biogenesis and maturation. Consistent with this notion, the vacuole-targeted late endocytic pathway marked by the small GTPase RabG3c (a Rab7 family member) is diverted toward the EHM during *P. infestans* infection of the solanaceous model plant *Nicotiana benthamiana* (Lu et al., 2012; Bozkurt et al., 2015). Upon activation, some PRRs are re-routed to the EHM through late endosomes (Bozkurt et al., 2015). However, it is unknown whether these PRRs are active in signaling or trapped at the haustorial interface by the pathogen in order to prevent their recycling back to cell surface, thus helping to suppress the host immune response.

Differential rerouting of the early endosomes (marked by Rab5), but not late endosomes (marked by RabG3f) towards the EHM has been observed in *Arabidopsis* leaves infected by two different oomycete pathogens (Lu et al., 2012). In contrast, the early endosomal marker Rab5 is excluded from the EHM engulfing the hemibiothrophic fungal pathogen *Colletotrichum higginsianum* (Inada et al., 2016)*.* These findings further highlight that EHM composition varies in different pathosystems, which could be due to the divergent strategies employed by pathogens to manipulate the EHM to support virulence. In support of this notion, several host-translocated RXLR effectors of *Phytophthora* accumulate and probably target the EHM (Bozkurt et al., 2011; Wang et al., 2019) (see poster), but how they reconfigure the EHM for the benefit of the pathogen remains to be elucidated.

Interestingly, REM1.3 and RabG3C label only about half of the EHM enveloping the *P. infestans* haustoria, suggesting that the EHM is a dynamic interface that undergoes maturation (Bozkurt et al., 2014, 2015). In agreement with this notion, *Arabidopsis* PLASMODESMATA-LOCATED PROTEIN 1 (PDLP1) localizes only to the non-encased EHM of the oomycete pathogen *H. arabidopsidis* (Caillaud et al., 2014). Thus, it is possible that the EHM is modified gradually, starting from the initial haustoria formation to its subsequent maturation and ultimate encasement, and pathogens could actively manipulate this process through host-translocated effectors.

## Diversion of autophagy machinery to the pathogen interface

Autophagy is a conserved eukaryotic trafficking process, in which cellular components and microbes are removed or relocated after engulfment in vesicular double-membrane-enclosed structures called autophagosomes (Lamb et al., 2013; Dagdas et al., 2018). Interestingly, selective forms of autophagy are induced at the perimicrobial interfaces in both plant and metazoan cells to counteract pathogen invasion (Thurston et al., 2012; Choi et al., 2014; Haldar et al., 2014; Schmuckli-Maurer et al., 2017; Wacker et al., 2017; Dagdas et al., 2018; Real et al., 2018). In plants, a defense-related autophagy machinery comprising the autophagy cargo receptor NBR1 (also known as Joka2) and the core autophagy adaptor ATG8 (ATG8CL isoform) target the EHM during *P. infestans* infection (see poster) (Dagdas et al., 2016). The pathogen counteracts this by deploying an RXLR effector called PexRD54. PexRD54 antagonizes NBR1 function by outcompeting it for ATG8CL binding, thereby neutralizing the defense-related autophagy at the haustorial interface (Dagdas et al., 2016, 2018). Thus, the autophagy machinery appears to participate in complex immune functions at perimicrobial membrane interfaces.

Unlike the many effectors of metazoan parasites that inhibit autophagy, PexRD54 stimulates formation of autophagosomes that accumulate at the haustorial interface (see poster). Why this is the case and what cargoes these autophagosomes carry remains uncharacterized. One hypothesis is that PexRD54 co-opts the host autophagy machinery as a molecular sink to absorb nutrients through the haustorial interface.

## Organelle trafficking to the pathogen interface

Early work showed that some plant organelles accumulate around the haustorial interface (Heath et al., 1997; Koh et al., 2005) (see poster). However, the mechanisms by which organelles are recruited to pathogen interface and how they function at these sites are unknown. Positioning the plant endomembrane system (nucleus, ER, Golgi and secretory vesicles) around the haustorial interface is considered to aid localized deployment of defense-related compounds (Schmelzer, 2002; Underwood and Somerville, 2008). Interestingly, the ER surrounding fungal haustoria in *Arabidopsis* has a different morphology from the remainder of the ER network, for example by exhibiting swollen tubes (Micali et al., 2011). Altered ER morphology correlates with restricted intra-luminal ER transport (Tolley et al., 2008). These changes in ER morphology could be possibly triggered by pathogens to counteract the focal deployment of secretory components to the haustorial interface.

Intriguingly, host mitochondria have also been reported to accumulate around the EHM during fungal invasion of barley (Kunoh and Ishizaki, 1973; Micali et al., 2011; Fuchs et al., 2016). Although how and why mitochondria are targeted to the haustorial interface is unknown, electron microscopy images revealed intimate interactions between mitochondria and the EHM, such as membrane fusions (Kunoh and Ishizaki, 1973). Likewise, chloroplasts also accumulate at the haustorial interface and form tubular extensions embracing the EHM (Toufexi et al., 2019 preprint). Notably, the chloroplast photorelocation protein CHLOROPLAST UNUSUAL POSITIONING 1 (CHUP1) is required for the perihaustorial positioning of chloroplasts and immunity against *P. infestans* (Toufexi et al., 2019preprint)*.* These findings implicate chloroplasts in plant immunity, but the exact defense-related functions of perihaustorial chloroplasts remain to be elucidated.

## Similarities between plant–pathogen and animal–parasite interfaces

The differences in EHM composition compared to the plasma membrane are reminiscent of the perimicrobial membrane interfaces that engulf metazoan parasites (Haldar et al., 2006). Intracellular mammalian parasites typically deploy a variety of effector proteins to divert the trafficking of Rab GTPases to the pathogen interface (Asrat et al., 2014). Interestingly, these Rab GTPases include Rab5 and Rab7 family proteins, which are also found to localize to the EHM of filamentous plant pathogens as discussed above. The host-derived membranes that engulf *Salmonella enterica* are marked by Rab5, whereas Rab7 is recruited during later stages of infection (Drecktrah et al., 2007). Such a stepwise maturation of the perimicrobial membrane interfaces could also be the underlying reason for partial labelling of the EHM with Rab7 (∼50%) we observed during *P. infestans* infection (Bozkurt et al., 2015). Strikingly, a time-dependent accumulation of Rab7 also occurs during maturation of the *Leishmania*-containing parasitophorous vacuole membrane (PVM) in mammalian cells, where Rab7 labels 70% of the PVMs within 30 min after infection and reaching complete coverage within 48 h (Courret et al., 2002). Interestingly, the early endosomal marker Rab5 was found to be excluded from *Leishmania* PVM in mammalian cells (Courret et al., 2002), which is similar to what is found for the *C. higginsianum* (hemibiotrhopic fungus) EHM in *Arabidopsis* (Inada et al., 2016). However, there are also differences in Rab requirement, as *Mycobacterium* phagosomal compartments in mammalian cells are Rab5 positive but lack Rab7 in mammalian cells (Via et al., 1997), which is similar to the EHM of biotrophic filamentous plant pathogens infecting *Arabidopsis* (Inada et al., 2016).

Another similarity between plant–pathogen and mammalian–parasite interfaces is the induction of autophagy responses that are directed towards the pathogens, which are contained in modified phagosomal compartments, similar to recent observations with *P. infestans* (Dagdas et al., 2016, 2018). For instance, components of mammalian autophagy machinery such as ATG8 (the LC3/GABRAP family in mammalian cells) as well as the autophagy cargo receptors p62 (also known as SQSTM) and NBR1 target the peri-microbial membrane interface engulfing the *Plasmodium* parasite (Schmuckli-Maurer et al., 2017; Wacker et al., 2017; Real et al., 2018) (see poster). Interestingly, similar to the *P. infestans* RXLR effector PexRD54, one of the PVM-embedded plasmodium effector proteins, called UIS3, binds to the mammalian ATG8 isoform LC3 to avoid being degraded by autophagy (Real et al., 2018). Similar to antagonistic relationship between PexRD54 and the plant autophagy receptor NBR1 that occurs at the EHM, UIS3 outcompetes the mammalian autophagy cargo receptors for ATG8 (LC3) binding at the PVM (Real et al., 2018). Plant NBR1 has a similar domain architecture and shares functional features of the mammalian autophagy receptors NBR1 and p62 (Svenning et al., 2011). It is not clear whether these autophagy cargo receptors convergently evolved to counteract microbial penetration of host cells. Nevertheless, it appears that both plant and mammalian parasites have developed similar strategies to disarm host cargo receptors at the pathogen interface.

## Conclusions and outlook

Despite the fact that phylogenetically diverse filamentous pathogens have convergently evolved the capacity to form haustoria and trigger the EHM interface with their plant hosts, there are some common principles. One common strategy employed by filamentous pathogens for successful invasion of the host cells is the reprogramming of host membrane trafficking pathways to avoid destruction by the host cellular defenses and facilitate efficient uptake of nutrients and possibly effector delivery. In addition, there are striking similarities in the processes that accommodate pathogens between plants and animals, some of which could possibly have originated from the ancestral eukaryotic cell. Future studies and emerging experimental systems, such as the fast-forward cell biology depicted in the poster, will help to further determine commonalities and differences across pathosystems and address pertinent questions about the haustorial interface. What are the mechanisms underlying the biogenesis and functions of the EHM? Does immune signaling take place at the pathogen interface? To what extent do pathogens manipulate the EHM for their own benefit? Does the EHM content differ depending on pathogen infection style? What are the origins and contents of the vesicles observed at the EHMX? Why and how do organelles traffic to the haustorial interface? How are nutrients and effectors transported across the haustorial interface? How is the autophagy machinery recruited to haustorial interface and how does it contribute to plant immunity? Answering these questions will further unveil the complex molecular and cellular processes that take place at the haustorial interface.
